# The combined effect of green tea and α-glucosyl hesperidin in preventing obesity: a randomized placebo-controlled clinical trial

**DOI:** 10.1038/s41598-021-98612-6

**Published:** 2021-09-24

**Authors:** Ren Yoshitomi, Mao Yamamoto, Motofumi Kumazoe, Yoshinori Fujimura, Madoka Yonekura, Yasuyo Shimamoto, Akari Nakasone, Satoshi Kondo, Hiroki Hattori, Akane Haseda, Jun Nishihira, Hirofumi Tachibana

**Affiliations:** 1 Division of Applied Biological Chemistry, Department of Bioscience and Biotechnology, Faculty of Agriculture, Kyushu University, 744 Motooka, Nishi-ku, Fukuoka, 819-0395 Japan; 2 Agriculture and Biotechnology Business Division, Toyota Motor Corporation, Aichi, Japan; 3 Department of Medical Management and Informatics, Hokkaido Information University, Hokkaido, Japan

**Keywords:** Health care, Weight management

## Abstract

Green tea, a widely consumed beverage in Asia, contains green tea catechins effective against obesity, especially epigallocatechin-3-*O*-gallate (EGCG), but must be consumed in an impractically huge amount daily to elicit its biological effect. Meanwhile, citrus polyphenols have various physiological effects that could enhance EGCG functionality. Here we investigated the antiobesity effect of a combination of EGCG and α-glucosyl hesperidin, a citrus polyphenol, at doses that have not been previously reported to exert antiobesity effects by themselves in any clinical trial. In a randomized, placebo-controlled, double-blinded, and parallel-group-designed clinical trial, 60 healthy Japanese males and females aged 30–75 years consumed green tea combined with α-glucosyl hesperidin (GT-gH), which contained 178 mg α-glucosyl hesperidin and 146 mg EGCG, for 12 weeks. Physical, hematological, blood biochemical, and urine examinations showed that GT-gH is safe to use. At week 12, GT-gH prevented weight gain and reduced body mass index (BMI) compared with the placebo. Especially in those aged < 50 years, triglyceride and body fat percentage decreased at week 6, visceral fat level and body fat percentage decreased at week 12; body weight, BMI, and blood LDL/HDL ratio also decreased. In conclusion, taking GT-gH prevents weight gain, and the antiobesity effect of GT-gH was more pronounced in people aged < 50 years.

## Introduction

Although the concept of complementary and alternative medicine (CAM) has been in existence for a long time, there are some similarities with Western medical perspectives^1^. Nutrition and supplements, some of the most popular and important CAM approaches, have great effects on our health, such as in patients with diabetes and hypertension^2–5^. However, some CAMs may not have the effects that were previously thought to be effective^6^. Therefore, recently, new CAM approaches have been implemented by combining food ingredients.

Overweight and obesity are medical conditions characterized by unusual or excessive fat accumulation that lead to diseases such as diabetes, hyperlipidemia, and hypertension. The current coronavirus disease 2019 (COVID-19) pandemic has led to economic shutdown and school and district closures worldwide, affecting the people mentally (e.g., stress) and physically (e.g., overeating and lack of exercise); thus, weight gain cases have increased globally^7,8^.

One of the most widely consumed beverages in Asia is green tea, which is derived from the tea plant *Camellia sinensis*. Green tea has several physiological functions, including anti-inflammatory^9^, antibacterial^10^, antiangiogenic^11^, antioxidant^12^, antiviral^13^, and neuroprotective effects^14^. Epigallocatechin-3-*O*-gallate (EGCG), which is the major catechin in green tea, is the most abundant polyphenol in tea leaves and reportedly exerts anticancer^15^, anti-inflammatory^16^, antidiabetic^17^, antiatherosclerotic^18^, and fat- and weight-reducing effects^19^. However, a clinical trial revealed that the antiobesity effect is not prominent unless EGCG is consumed in very high quantities (at least 300 mg/day)^20^.

According to the Osaki cohort study, consumption of both green tea and citrus fruits daily can reduce the incidence of cancer^21^. Metabolic profiling-based data mining showed that eriodictyol, a type of citrus polyphenol, enhances the anticancer effect of EGCG^22^. In mice fed with high-fat/high-sucrose diet, green tea and eriodictyol combination prevented weight gain and reduced low-density lipoprotein (LDL) cholesterol levels^23^. These studies suggest that the function of EGCG can be enhanced by simultaneous intake of citrus polyphenols. However, the combined effect of green tea and citrus-derived polyphenols has not been verified in human trials.

Therefore, this study aimed to evaluate the antiobesity effect of a combination of EGCG (146 mg/day) and α-glucosyl hesperidin (178 mg/day), a type of citrus polyphenol, at doses that have not been previously reported to exert antiobesity effects by themselves in any clinical trial.

## Results

### Subjects

**Figure 1:**
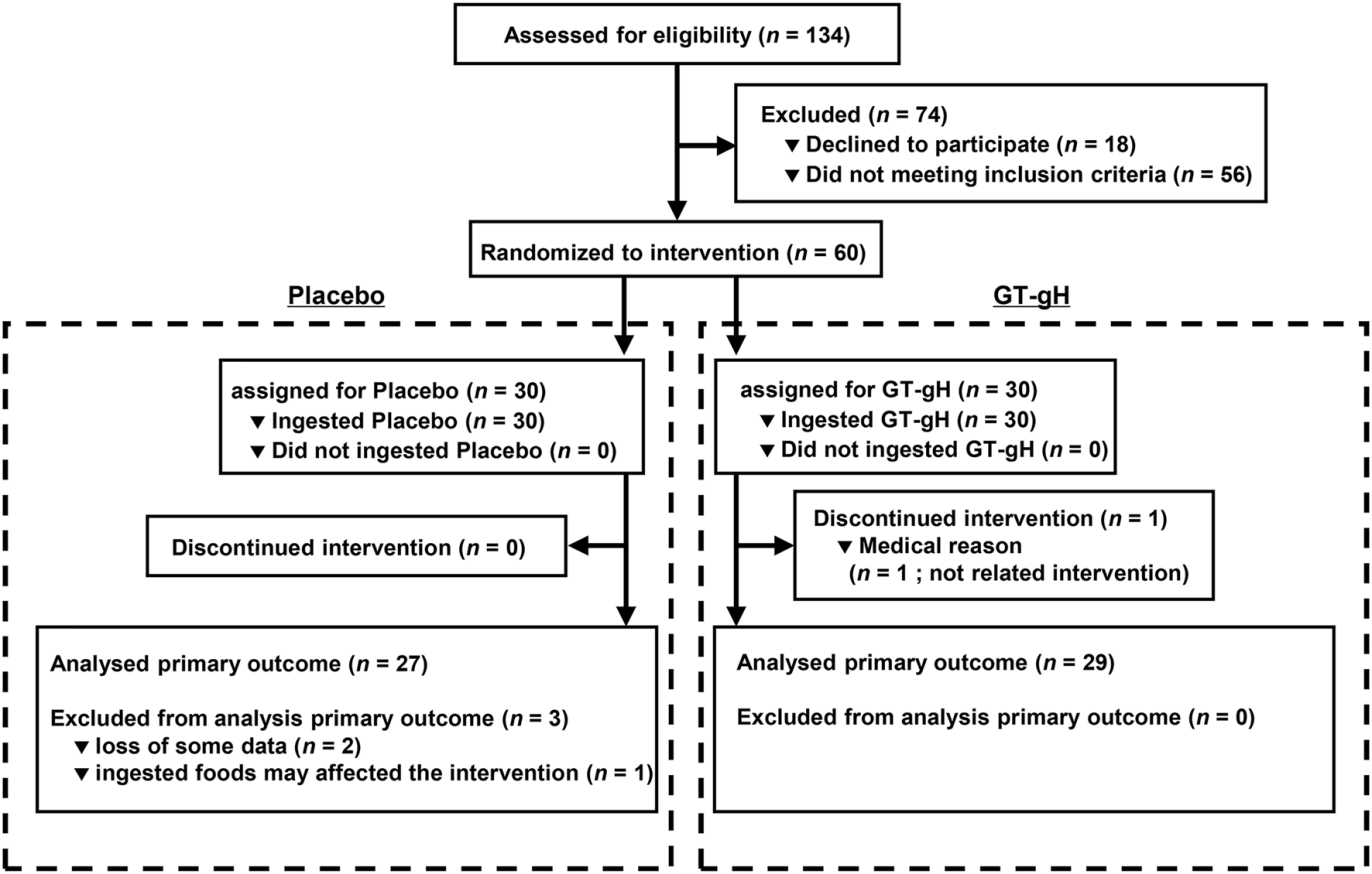
Transition chart of the intervention participants.

Of the 134 subjects, 60 Japanese males and females who were 30–75 years old and had BMI of 23–30 kg/m^2^ and LDL cholesterol levels of 100–140 mg/dL were eligible to participate. Table 1 shows the breakdown of these subjects, and Fig. 1 presents a flow chart of the number of subjects per group in the study. A subject from the GT-gH group discontinued the study because of the relapse of duodenal ulcer after week 6. However, the responsible physician confirmed that the medical incident was not related to this study. Therefore, 59 subjects (30 in the placebo group and 29 in the GT-gH group) completed the study. Two participants were excluded from the analysis because of missing values for the primary endpoint (the placebo group), and another participant was excluded because of the intake of brown rice, which could affect the overall analysis (the placebo group). Therefore, three participants were excluded from the efficacy analysis (Fig. 1).

**Table 1:** Baseline characteristics of participants. Data shows Means ± SD. GT-gH, green tea with α-glucosyl hesperidin. *P* value were calculated by independent two-sample *t* test.

Variable	Placebo (*n* = 30)	GT-gH (*n* = 30)	*P* value
Age (years)	53.43 ± 9.87	53.87 ± 9.64	0.864
Male [n (%)]	14 (47%)	16 (53%)	–
Female [n (%)]	16 (53%)	14 (47%)	–
Hight (cm)	161.98 ± 8.19	163.75 ± 8.29	0.408
Weight (kg)	66.70 ± 8.73	67.84 ± 8.09	0.601
Fat (%)	30.33 ± 6.89	29.47 ± 7.98	0.659
BMI (kg/m^2^)	25.25 ± 1.88	25.25 ± 1.88	0.857

### Safety

In the safety analysis, we examined the vital signs (SBP, DBP, and pulse rate). In the placebo group, SBP was significantly higher at week 12 than at week 0. Within each group, hematological tests (WBC, RBC, hemoglobin, hematocrit, platelets) and liver function (AST, ALT, γ-GTP, ALP, and LDH) and renal function (BUN, CRE, and UA) were significantly different. In blood glucose analysis (fasted blood glucose and HbA1c), HbA1c was higher at week 12 than at week 0 in both groups. However, at weeks 6 and 12, the GT-gH group had a significantly lower HbA1c value than the placebo group.The urine tests (pH, glucose, protein, occult blood, urobilinogen, and ketone bodies) showed that at week 6, the GT-gH group had a significantly lower urine pH than the placebo group (Table S1). Nonetheless, all values in Table S1. were within normal limits. Thus, GT-gH was safe to use for 12 weeks.

### Antiobesity effect of GT-gH

**Figure 2:**
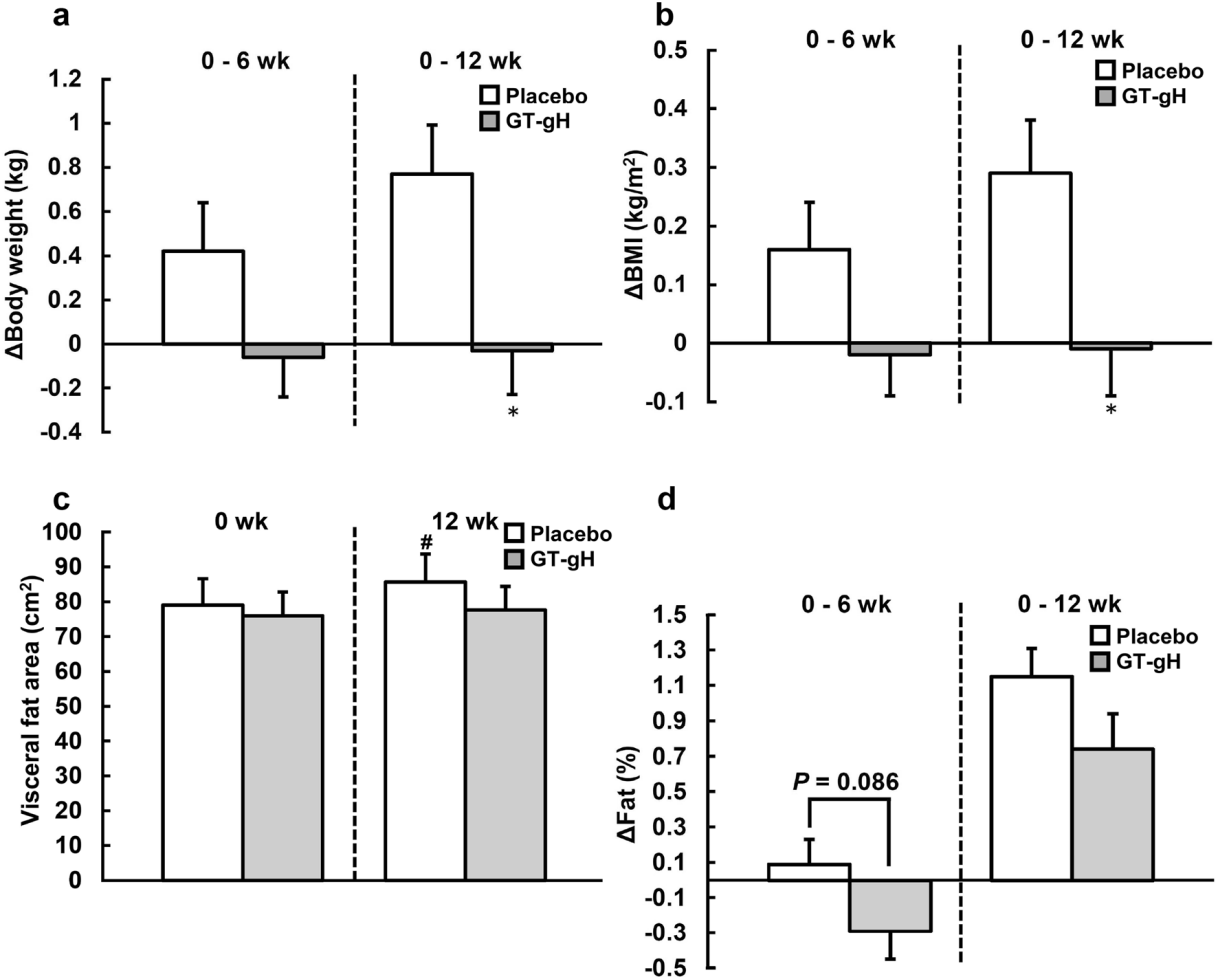
Diachronic change in visceral fat area, body weight, BMI and fat in the placebo (n = 27) and GT-gH (n = 29) groups. (**a**) Body weight, (**b**) BMI and (**d**) fat were measured by a dual-frequency body composition analyzer, and the changes in their values were analyzed by an independent two-sample *t*-test. (**c**) The visceral fat area was measured by PET-CT and Fat Checker. For between-group comparisons, the values were analyzed using an independent two-sample *t*-test. *P < 0.05 (vs. placebo). For within-group comparisons, the values were analysed by a paired *t*-test. #*P* < 0.05 (vs. week 0). Grey bars represent GT-gH, and open bars represent placebo. Data are shown as means ± SE. BMI, body mass index; GT-gH, green tea with α-glucosyl hesperidin; PET-CT, positron emission tomography with computed tomography.

After 12 weeks of GT-gH intake, changes in body weight and BMI were significantly different between the groups (body weight: *p* = 0.010, BMI: *p* = 0.014), indicating that the increase in body weight and BMI was considerably prevented in the GT-gH group (Fig. 2a,b).

Compared with the baseline, the visceral fat area in the placebo group significantly increased after 12 weeks of consumption (*p* = 0.038), but that in the GT-gH group demonstrated no significant difference (*p* = 0.548). Therefore, the increase in visceral fat area was suppressed in the GT-gH group (Fig. 2c). Meanwhile, changes in fat percentage showed no significant difference between the groups at week 12 but tended to significantly differ at week 6 (Fig. 2d).

### Subgroup analysis of subjects below 50 years old

**Figure 3:**
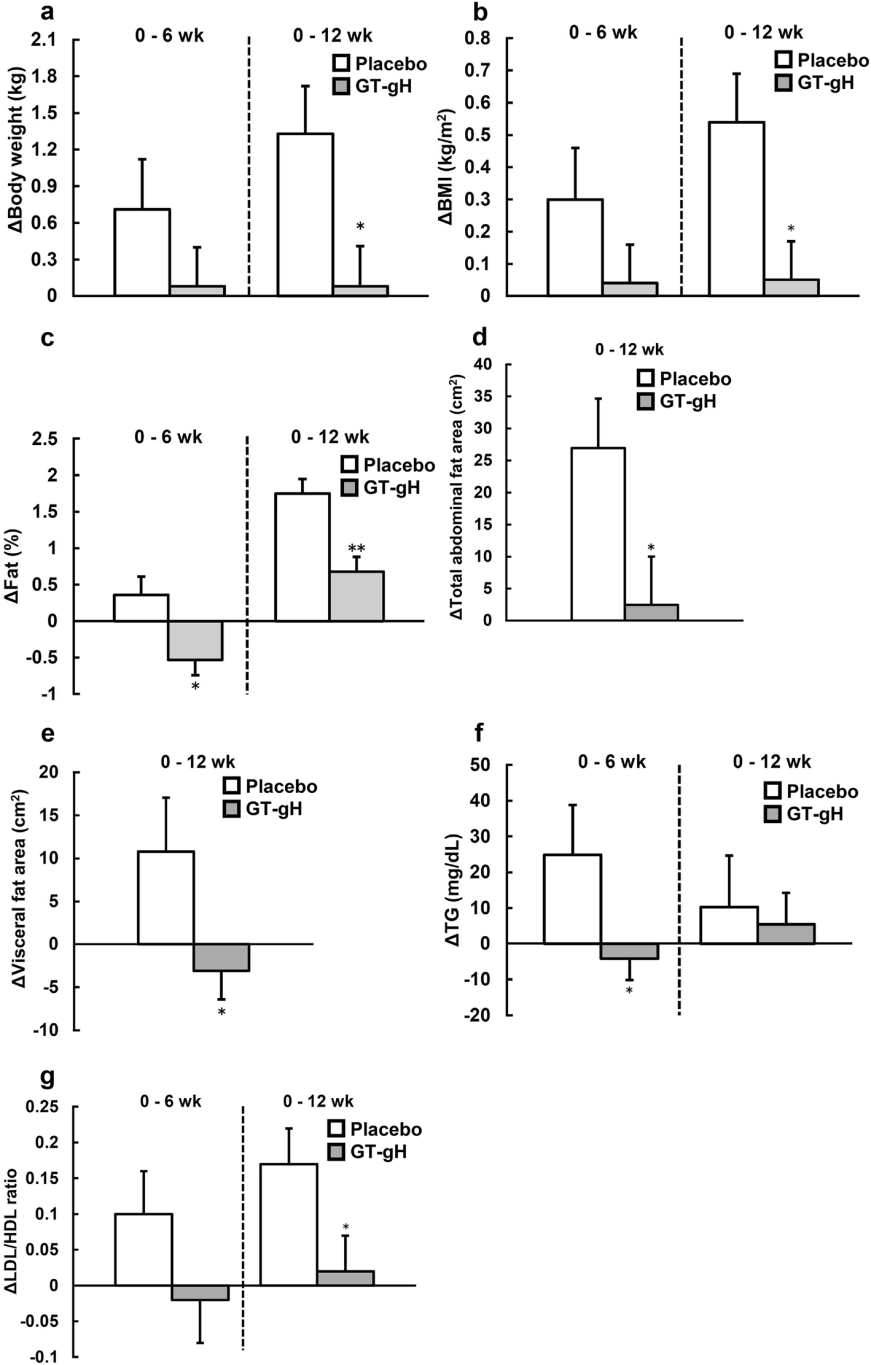
Subgroup analysis of the change amount of obesity-related parameters in subjects below 50 years old in the placebo (n = 10) or GT-gH (n = 13) groups. (**a**) Body weight, (**b**) BMI, and (**c**) fat were measured by a dual-frequency body composition analyzer. (**d**) Total abdominal fat area and (**e**) visceral fat area were measured by PET-CT and Fat Checker. (**f**) TG was measured by free-glycerol elimination method, (**g**) LDL/HDL ratio was measured by selective solubilization method and selective suppression method. (**a**–**g**) were analyzed by independent two-sample t-test. *p < 0.05, **p < 0.01 (vs. placebo). Gray bars represent GT-gH, and open bars represent placebo. Data are shown as means ± SE. *GT-gH* green tea with α-glucosyl hesperidin; *LDL/HDL ratio* ratio of low-density lipoprotein to high-density lipoprotein; *TG* triglyceride.

To perform exploratory data analysis, the subgroup analysis was conducted according to age (< 50 years: 10 in the placebo group and 13 in the GT-gH group; ≥ 50 years: 17 in the placebo group and 16 in the GT-gH group). In the GT-gH group, significant differences were observed in visceral fat area (week 12: *p* = 0.0498), total abdominal fat area (week 12: *p* = 0.037), TG (week 6: *p* = 0.0498), LDL/HDL ratio (week 12:* p* = 0.038), body weight (week 12: *p* = 0.022), body fat percentage (weeks 6 and 12: *p* = 0.013 and *p* = 0.001, respectively), and BMI (week 12:* p* = 0.021) (Fig. 3). Thus, the antiobesity effect of GT-gH was more enhanced in people below 50 years old.

## Discussion

This study was conducted from June to October of 2020. During this period, the COVID-19 pandemic had led people to different unhealthy habits such as lack of exercise caused by restrictions on going out, and excessive calorie intake caused by overeating. This experiment found that weight gain was prevented in the GT-gH group compared with that in the placebo group.

According to several studies, green tea and EGCG have beneficial effects on weight. For example, a randomized controlled crossover trial of males (age, 40–69 years; BMI, ≥ 28 and ≤ 38 kg/m^2^) who consumed green tea for 6 weeks showed reduction in body weight^24^. In a randomized, double-blind, crossover- and placebo-controlled clinical trial, females who consumed green tea extract for 6 weeks showed increase in leptin levels and decrease in LDL cholesterol levels^25^. However, high EGCG levels (856.8 and 431.5 mg) were used daily in the abovementioned trials. In a meta-analysis, the 12-week intake of beverages containing high tea catechins (> 539.7 mg of tea catechins/day) reduced visceral fat and body weight in healthy Japanese subjects (BMI > 25 and < 30 kg/m^2^)^26^. In addition, α-glucosyl hesperidin did not exert antiobesity effects even at 500 mg in a human clinical trial^27^. In contrast, the present study revealed that 146 mg of EGCG combined with 178 mg of α-glucosyl hesperidin exerted antiobesity effects. In the adult population of the European Union, the average daily intake of EGCG from the consumption of green tea extract is 90–300 mg/day^28^; therefore, the amount of EGCG contained in GT-gH is within the range of daily intake. Although the amounts of EGCG and α-glucosyl hesperidin in our study were lower than the effective amounts causing antiobesity effects, the amount of EGCG contained in GT-gH was safe to be consumed daily.

Citrus polyphenols, such as eriodictyol and hesperidin, have various physiological effects, including antioxidant^29^, anti-inflammatory^30^, and hypertension-inhibitory effects^31^. The soluble hesperidin derivative, α-glucosyl hesperidin, which is used as a food additive, is considerably more water-soluble than hesperidin. α-glucosyl hesperidin converts into hesperetin in the gut, and hesperetin could be metabolized to eriodictyol in the liver and kidney^32^. Thus, α-glucosyl hesperidin is thought to act as hesperetin or eriodictyol in vivo.

The combined effects of food components have been extensively analyzed in cell and animal experiments. For example, the combination of green tea and eriodictyol reduced body weight and LDL cholesterol levels in obesity-induced mice^23^. Furthermore, the combination of resveratrol and quercetin attenuated obesity and modulated gut microbiota in rats^33^. However, most of the studies demonstrated murine and cellular experiments. The present study revealed the combined effects of food components that have rarely been clarified in clinical practice, thereby providing valuable information in clinical nutrition.

The safety assessment results of vital signs (SBP on arrival, DBP on arrival, pulse rate), general hematology (WBC, RBC, hemoglobin, hematocrit, and platelet), liver function (AST, ALT, γ-GTP, ALP, and LDH), renal function (BUN, CRE, and UA), and blood glucose (fasting blood glucose, HbA1c) were not significantly different between the placebo and GT-gH groups. Statistical analysis revealed significant differences in some of these items between the groups, but all of the changes were within normal limits and did not pose a clinical problem. No serious or medically problematic adverse reactions were observed within the study period. As for the adverse events, we found no clinically problematic clinical findings or abnormal changes in laboratory values. Therefore, continuous intake of GT-gH for 12 weeks is safe.

This study found that GT-gH suppressed weight gain and BMI increase. Furthermore, stratified analysis based on the age of 50 showed that not only body weight and BMI but also visceral fat level, body fat percentage, and blood LDL/HDL ratio decreased in subjects aged < 50 years.

In conclusion, GT-gH intake suppressed weight gain. However, significant differences in fat (weeks 6 and 12), visceral fat area (week 12), total abdominal fat area (week 12), TG (week 6) and LDL/HDL ratio (week 12) were found only in subjects under 50 years.

EGCG exerts various physiological effects, such as anticancer and anti-inflammatory effects, via the 67 kDa laminin receptor (67LR); if the 67LR expression decreases, the effect of EGCG is inhibited^34–36^. Therefore, the expression level of 67LR may be different between those who aged ≥ 50 years and those who aged < 50 years. However, further investigation is required to determine the reason for the greater effect of GT-gH in people aged < 50 years.

## Methods

### Study design

In this placebo-controlled, randomized, double-blind, parallel-group study, participants consumed placebo (barley tea powder, Mitsui Norin) or green tea combined with α-glucosyl hesperidin (GT-gH) (Mitsui Norin) once daily for 12 weeks. The participants consumed two packets (2 × 6.8 g) of placebo (barley tea powder) or GT-gH (146 mg EGCG and 178 mg α-glucosyl hesperidin daily). Based on the previous clinical trials that demonstrated visceral fat area-reducing effects of green tea with high catechin content, we set the desired group difference in visceral fat area change associated with GT-gH intake at 8.0 cm^2^^26^. Assuming that the standard deviation of the change in visceral fat area in each group was 10 cm^2^^37–39^, the number of subjects required to guarantee the results of analysis using independent two-sample *t*-test at a significance level of 5% two-sided and 80% power was 27 subjects in each group. We estimated that the rate of study discontinuation and exclusion would be within 10%, and set a target enrollment of 30 subjects per group.

### Participants

This study included 60 healthy Japanese males and females who were between 30 and 75 years of age and were not regular green tea consumers. Their body mass index (BMI) and LDL cholesterol values were between 23 and 30 kg/m^2^ and between 100 and 140 mg/dL, respectively.

### Study foods and dietary restrictions

After a washout period of 1 week, participants consumed two packets (2 × 6.8 g) of placebo (barley tea powder) or GT-gH, dissolved in 200 mL of water or hot water, once daily for 12 weeks. On a daily basis, GT-gH, which is powdered green tea extract, contained 178 mg of α-glucosyl hesperidin and 146 mg of EGCG. The amounts of EGCG and α-glucosyl hesperidin were determined at amounts that have not been reported to have antiobesity effects. In the placebo food (barley powder), the barley extract was used as a substitute for green tea extract and α-glucosyl hesperidin. Both the placebo and GT-gH were manufactured in the same factory using the same production methods. The participants were informed that the tea powder was either a “tea powder with citrus” or a “tea powder without citrus” and that neither the test nor the placebo food had a citrus taste or aroma. They were not explained that the green tea was the experimental diet or that the barley tea was the placebo food. During the intervention period, we prohibited the intake of green tea, fresh mandarin oranges, and other healthy foods (especially those that affect lipid and glucose metabolism) and the excessive consumption of citrus fruit juices and citrus fruits and processed foods.

### Experimental procedure

The vital signs, body measurements, blood tests, and urine tests of the participants were assessed at weeks 0, 6, and 12. Abdominal CT was performed at weeks 0 and 12. Using the stratified permuted block method, we randomly categorized the subjects according to gender age, BMI, and LDL cholesterol into two groups as follows: placebo group and GT-gH group. Subjects will be randomized by a stratified replacement block method using gender, age structure, BMI and LDL-C as stratification factors. Randomization will be conducted by the person in charge of food allocation and an allocation table will be prepared. The allocation list will be stored as an electronic medium with a password in a locked place by the person in charge of food allocation. The allocation was disclosed after the study was completed, and the cases, data, and analysis methods were fixed. The change in the amount of visceral fat area was our primary endpoint, whereas the other obesity-related parameters were our secondary endpoints.

### Vital signs and body measurement

Systolic blood pressure (SBP), diastolic blood pressure (DBP), and pulse rate were measured using a digital automatic blood pressure monitor (Omron) at the time of visit at the Healthcare Center of Hokkaido Information University. For measuring the body weight, body fat percentage, BMI, lean body mass, and muscle mass, we used a dual-frequency body composition analyzer (Tanita).

### Blood tests

Blood was drawn using a blood collection tube. The process from blood collection to sample analysis was quick and without freezing. White blood cells (WBC), red blood cells (RBC), hemoglobin, hematocrit, and platelets were measured using an automated hematology analyzer (Sysmex) at Sapporo Clinical Laboratory. We also measured the LDL cholesterol, high-density lipoprotein cholesterol (HDL-C), TG, aspartate transaminase (AST), alanine transaminase (ALT), γ-glutamyl transpeptidase (γ-GTP), alkaline phosphatase (ALP), lactate dehydrogenase (LDH), blood urea nitrogen (BUN), creatinine (CRE), urine acid (UA), fasted blood glucose, and hemoglobin A1c (HbA1c) were measured at the same laboratory.

### Urinalysis

We collected urine in the morning of the test day and measured its pH by using a pH test paper at Sapporo Clinical Laboratory. We also used the test paper to determine the presence of sugar, protein, occult blood, urobilinogen, ketone bodies, and bilirubin in the urine.

### CT scan of the abdomen

The subjects fasted for 2 h before the CT scan, which was performed using Discovery ST Elite Performance (GE Healthcare) according to the protocol of Teishinkai Central CI Clinic. The subjects were placed in a prone position inside the machine, with fists raised and breathing stopped. The fourth center of the lumbar spine was imaged with a slice thickness of 10 mm (tube voltage, 120 kvp; tube current, 150 mA; window width, 350; and window level, 35). The average radiation dose was 5 mSv per session. The images were analyzed using Fat Checker (J-MAC SYSTEM) to calculate the visceral fat area, total abdominal fat area, and subcutaneous abdominal fat area.

### Statistical analysis

We used the independent two-sample *t*-test to compare the baseline characteristics of the participants between groups, the vital signs, blood analysis, and urinalysis (pH) between groups, and the change amounts of obesity-related parameters (visceral fat area, total abdominal fat area, subcutaneous fat area, TG, and LDL/HDL) between groups. For within-group comparisons (week 0 vs. week 6 and week 0 vs. week 12), the values were analyzed by paired *t*-test^39^. For urinalysis (sugar, protein, occult blood, urobilinogen, ketone bodies), the differences between Placebo and GT-gH or between week 0 and week 6, 12 were analyzed with chi‐square test. All descriptive statistics and statistical hypothesis testing were performed using SPSS (version 25, IBM).

### Ethical approval

This study is in compliance with the Declaration of Helsinki and the Ethical Guidelines for Medical and Health Research Involving Human Subjects (Ministry of Education, Culture, Sports, Science and Technology and Ministry of Health, Labor, and Welfare). The TOYOTA MOTOR CORPORATION Research Ethics Review Committee, the Ethics Committee for Human Health of Hokkaido Information University, and the Ethics Committee of Kyushu University reviewed and approved in advance the feasibility and ethical and scientific validity of the clinical trial. All subjects provided written informed consent before participating in the study. Before we started this clinical trial, we registered the summary of our experiment in the University Hospital Medical Information Network (UMIN) (08/04/2020 UMIN ID: UMIN000040109, title: Effect of Daily Ingestion of tea containing citrus ingredients on abdominal visceral fat area reduction: A Randomized, Double-Blind, Placebo-Controlled, Parallel Group Comparison Study).

## Supplementary Information

Supplementary Information.

## Data Availability

Data described in the manuscript will not be made available because approval has not been granted by subjects for data upload.
