# Supplementary material for: The combined effect of green tea and α-glucosyl hesperidin in preventing obesity: a randomized placebo-controlled clinical trial
Source: Sci Rep. 2021 Sep 24;11:19067. doi: 10.1038/s41598-021-98612-6 (PMC8463579; doi:10.1038/s41598-021-98612-6)
Supplement: Supplementary file 1 — Supplementary Information. [file 41598_2021_98612_MOESM1_ESM.docx]

**The combined effect of green tea and α-glucosyl hesperidin in preventing obesity: a randomized placebo-controlled clinical trial**

**Ren Yoshitomi^1, #^, Mao Yamamoto^1, #^, Motofumi Kumazoe^1^, Yoshinori Fujimura^1^,** **Madoka Yonekura^2^, Yasuyo Shimamoto^2^, Akari Nakasone^2^, Satoshi Kondo^2^, Hiroki Hattori^3^, Akane Haseda^3^, Jun Nishihira^3^, and Hirofumi Tachibana^1*^**

^1^Division of Applied Biological Chemistry, Department of Bioscience and Biotechnology, Faculty of Agriculture, Kyushu University, Fukuoka, Japan

^2^Agriculture & Biotechnology Business Division, TOYOTA MOTOR CORPORATION, Aichi, Japan

^3^Department of Medical Management and Informatics, Hokkaido Information University, Hokkaido, Japan

^#^These authors contributed equally to this work.

Supplemental Material File Listing:

Table S1

Corresponding author

Hirofumi Tachibana, 744 Motooka, Nishi-ku, Fukuoka 819-0395, Japan; Tel and Fax: (+81) (92) 802-4749

E-mail: [tatibana@agr.kyushu-u.ac.jp](mailto:tatibana@agr.kyushu-u.ac.jp)

**Table S1. Safety analysis before and after 6 weeks and 12 weeks of intervention in patients consuming placebo and green tea with α-glucosyl hesperidin (GT-gH).**

| **Variable** | **Placebo** | | | **GT-gH** | | |
| --- | --- | --- | --- | --- | --- | --- |
|  | **0 wk** | **6 wk** | **12 wk** | **0 wk** | **6 wk** | **12 wk** |
| **Vital sign** | | | | | | |
| SBP (mmHg) | 118.87±18.85 | 121.83±16.04 | 123.80±19.06* | 121.93±17.81 | 122.03±14.93 | 123.72±17.54 |
| DBP (mmHg) | 80.87±10.39 | 78.53±9.85 | 82.90±11.31 | 82.43±13.30 | 81.63±13.21 | 83.79±12.16 |
| Pulse rate (bpm) | 74.50±11.18 | 73.87±13.11 | 72.33±12.62 | 75.47±10.96 | 74.33±10.29 | 73.93±11.48 |
| **Hematologic parameters** | | | | | | |
| WBC (cells×10^3^/µL) | 5.24±1.25 | 5.66±1.31** | 5.42±1.40 | 4.94±0.98 | 5.10±0.98 | 4.90±1.14 |
| RBC (cells×10^4^/µL) | 471.97±44.50 | 463.90±40.01** | 477.63±42.79 | 472.20±47.55 | 467.33±43.20 | 483.17±43.76 |
| Hemoglobin (g/dL) | 14.11±1.38 | 13.88±1.41** | 14.27±1.54 | 14.17±1.44 | 14.00±1.42 | 14.53±1.29** |
| Hematocrit (%) | 42.59±3.31 | 41.74±3.27** | 43.08±3.67 | 42.57±3.81 | 41.86±3.94** | 43.52±3.61** |
| Platelets (cells×104/µL) | 26.93±6.46 | 26.79±6.30 | 28.26±6.68** | 25.29±6.52 | 25.23±6.42 | 25.66±6.59 |
| **Biochemical parameters of blood** | | | | | | |
| AST (IU/L) | 23.67±6.07 | 22.27±6.10 | 23.67±7.47 | 23.13±6.54 | 22.80±3.43 | 22.59±3.51 |
| ALT (IU/L) | 23.70±14.00 | 22.77±13.54 | 24.93±17.94 | 24.80±11.77 | 24.40±9.87 | 25.24±10.50 |
| γ-GTP (IU/L) | 29.93±18.97 | 31.80±19.99 | 33.10±22.27* | 39.80±50.01 | 42.20±54.01 | 38.90±35.81 |
| ALP (IU/L) | 192.17±48.20 | 182.87±40.15** | 191.27±45.77 | 197.70±52.30 | 203.20±54.75 | 203.86±53.35 |
| LDH (IU/L) | 193.30±22.52 | 190.77±23.94 | 191.43±22.68 | 188.77±23.68 | 191.60±24.08 | 188.86±23.25 |
| BUN (mg/dL) | 14.49±3.57 | 14.58±4.12 | 14.65±3.55 | 13.82±2.77 | 14.88±3.31* | 13.22±3.07 |
| CRE (mg/dL) | 0.84±0.18 | 0.84±0.18 | 0.82±0.18* | 0.84±0.17 | 0.85±0.16 | 0.82±0.15 |
| UA (mg/dL) | 5.55±1.22 | 5.74±1.35 | 5.63±1.37 | 5.34±1.29 | 5.69±1.25** | 5.50±1.31* |
| FBG (mg/dL) | 90.10±8.48 | 91.57±8.48 | 92.43±10.16 | 87.97±10.08 | 88.80±8.89 | 88.93±9.74 |
| HbA1c (%) | 5.45±0.28 | 5.45±0.27 | 5.59±0.31** | 5.32±0.24 | 5.27±0.20^##^ | 5.40±0.24*^, #^ |
| **Urinalysis** | | | | | | |
| Urine pH | 6.12±0.76 | 6.27±0.77 | 6.02±0.62 | 6.03±0.60 | 5.90±0.62^#^ | 6.10±0.76 |
| Sugar in urine  within baseline  out of baseline | 30  0 | 30  0 | 30  0 | 30  0 | 30  0 | 29  0 |
| Protein in urine  within baseline  out of baseline | 30  0 | 30  0 | 29  1 | 30  0 | 30  0 | 28  1 |
| Occult blood in urine  within baseline  out of baseline | 30  0 | 29  1 | 25  5 | 29  1 | 25  5 | 27  2 |
| Urobilinogen in urine  within baseline  out of baseline | 30  0 | 30  0 | 30  0 | 30  0 | 30  0 | 29  0 |
| Ketone bodies in urine  within baseline  out of baseline | 30  0 | 30  0 | 29  1 | 30  0 | 30  0 | 29  0 |

Data show means ± SD. The differences between 0 and 6–12 weeks were analysed using a paired *t*-test. **P* < 0.05, ***P* < 0.01 (*vs*. 0 week). Differences between placebo (n = 30) and GT-gH (n = 29−30) were analysed with an independent two-sample *t*-test. #*P* < 0.05, # # *P* < 0.01 (*vs*. placebo). For urinalysis (sugar, protein, occult blood, urobilinogen, ketone bodies), the differences between Placebo and GT-gH or between 0 wk and 6, 12 wk were analyzed with chi‐square test. ALP, alkaline phosphatase; ALT, alanine aminotransferase; AST, aspartate aminotransferase; BUN, blood urea nitrogen; CRE, creatinine; DBP, diastolic blood pressure; FBG, fasting blood glucose; γ-GTP, γ-glutamyl transpeptidase; GT-gH, green tea with α-glucosyl hesperidin; HbA1c, haemoglobin a1c; LDH, lactate dehydrogenase; RBC, red blood cells; SBP, systolic blood pressure; UA, uric acid; WBC, white blood cells.
